# A Case Report of a Uterine Smooth Muscle Tumor of Undetermined Malignant Potential (STUMP): A Diagnostic Dilemma and Management Challenges

**DOI:** 10.7759/cureus.58067

**Published:** 2024-04-11

**Authors:** Ban Hadi, Wasan Nori, Hajer Ibrahem, Zeena R Helmi

**Affiliations:** 1 Obstetrics and Gynecology, College of Medicine, Mustansiriyah University, Baghdad, IRQ; 2 Oral Medicine, College of Dentistry, Ashur University, Baghdad, IRQ

**Keywords:** oncology, goserelin acetate, hysterectomy, myomectomy, stump

## Abstract

Uterine smooth muscle tumors of undetermined malignant potential (STUMPs) are an uncommon kind of uterine smooth muscle tumor. It is distinguished by histopathologic and morphologic characteristics that are in between those of a benign leiomyoma and a malignant leiomyosarcoma. From a clinical standpoint, the clinical presentation of STUMP is similar to that of a fibroid. The diagnosis is usually confirmed after surgery.

Here, we report the case of a 39-year-old woman who experienced increased menorrhagia, back pain, and pressure sensations during the past six months. She had a well-defined, freely movable lump in her lower abdomen, measuring the size of a 22-cm uterus. The patient exhibited pallor, and her imaging scan showed the presence of an intramural posterior uterine solid mass indistinguishable from fibroid measuring 8.5 × 9 cm. Goserelin acetate (Zoladex 3.6 mg implant) was recommended for a duration of six months. The patient experienced a significant amelioration in menorrhagia and discomfort. However, no reduction in the size of the mass was observed. Myomectomy was made for the suspicion of a malignant transformation. The histology examination confirmed the diagnosis of a STUMP; a hysterectomy was undergone, and the procedure went smoothly. The patient was discharged home in good condition with instructions for long-term follow-up due to a risk of recurrence of about 7%. The lack of standardized and clear clinical and diagnostic criteria for STUMP adds challenges to their management.

## Introduction

This study aims to shed light on this rare tumor. Uterine smooth muscle tumors of uncertain malignant potential (STUMPs) are rare smooth muscle tumors of the uterus that cannot be categorized as benign or malignant according to their histological features [1]. They have unpredictable biological behavior and the potential for recurrence or metastasis. STUMPs account for around 1% of all uterine smooth muscle tumors. They lack definitive criteria to be classified as malignant [2]. Clinically, STUMPs may present as abnormal uterine bleeding, pelvic pain, and/or a mass resembling uterine fibroids. Their diagnosis is only confirmed after surgical removal and a histopathological exam [3]. Herein, we present the case of a 39-year-old lady who was managed as a case of fibroid that failed to respond to medical treatment. Later, the histopathological report confirmed STUMPs.

## Case presentation

A 39-year-old lady presented to our department complaining of menorrhagia, abdominal pain, and pelvic pressure symptoms for six months. She was G3P3A0 all by cesarean sections (C-section); her last delivery was four years ago, where tubal ligation was done at the time of the C-section. On examination, a white female with an average body build, looking pale with normal vital signs, was noted. There was a palpable mass at her lower abdomen: tender, firm in consistency, freely mobile, and not attached to the skin or underlying structure. Its size reached 22 cm. Per vaginal examination, showed an anteverted enlarged uterus, with a normal-looking cervix and free adnexa.

**Figure 1:**
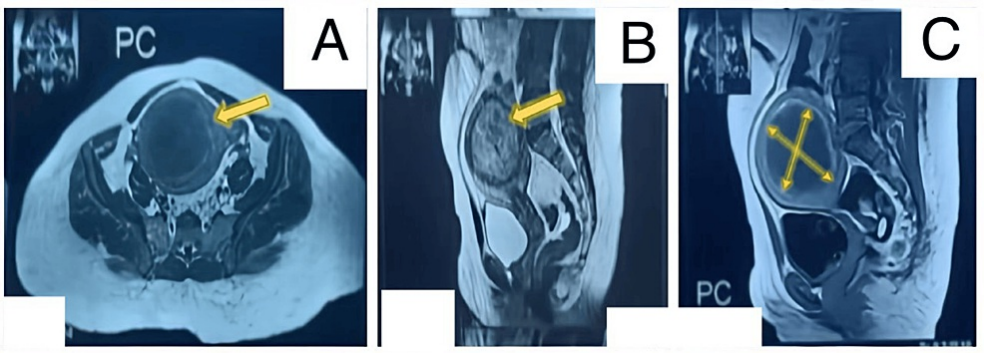
MRI with contrast (A) axial T1WI post-contrast section; (B) sagittal T2WI; (C) sagittal T1 post-contrast. The sections through the pelvis show a bulky size anteverted uterus with well-defined RT fundal intramural mass lesion (yellow arrows) measured (90x80 mm), showing heterogenous signal intensity in T2WI, showing minimal enhancement post-contrast administration causing indentation on the endometrium.

Hematological tests revealed severe anemia (7.6 g/dl.), but white blood cells and platelets were normal. Liver and renal function was unremarkable. Tumor markers (CA125, LDH) were negative, too. Imaging tests, including ultrasound and magnetic resonance imaging (MRI), confirmed a single well-defined big intramural posterior uterine fibroid measuring 8.5 ×9 cm, compressing a normal thin endometrium line. The ovaries and the bony pelvis were normal, with no ascites, no lymph nodes, and a clear cul-de-sac, see Figures 1A-1C. Diagnosing the condition and uterine myoma opted for medical treatment: goserelin acetate (Zoladex 3.6 mg implant, AstraZeneca, Cambridge, United Kingdom) was prescribed for six months with iron therapy to correct anemia. In the follow-up visits, there was considerable improvement in menorrhagia and pain. However, the size of the mass by abdominal examination remains the same. The decision for myomectomy was made based on the suspicion of a malignant transformation into leiomyosarcoma.

**Figure 2:**
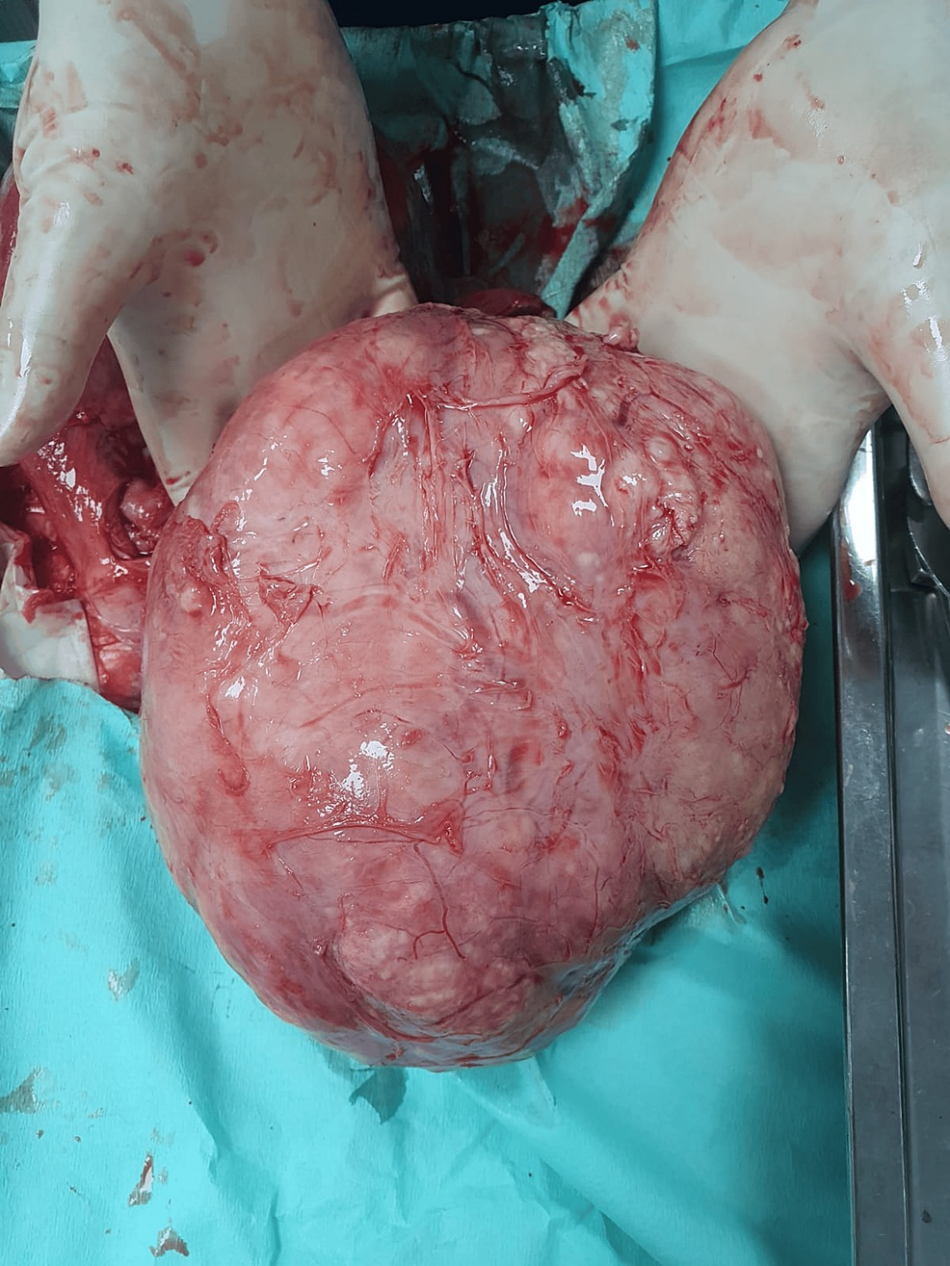
Macroscopic picture of a well-circumscribed, fleshy mass, measuring 9× 10 cm in the largest diameter

**Figure 3:**
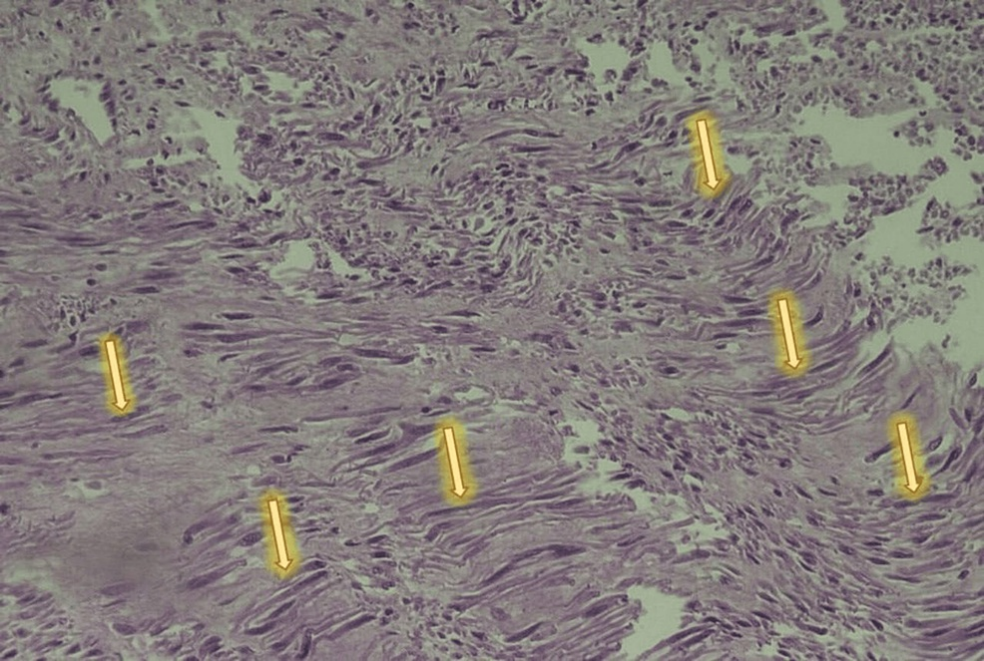
Photomicrograph of the lesion showing bundles of smooth muscle fibers with moderate nuclear atypia; some nuclei are hyperchromatic and some are irregular in the nuclear membrane H&E: 40X

**Figure 4:**
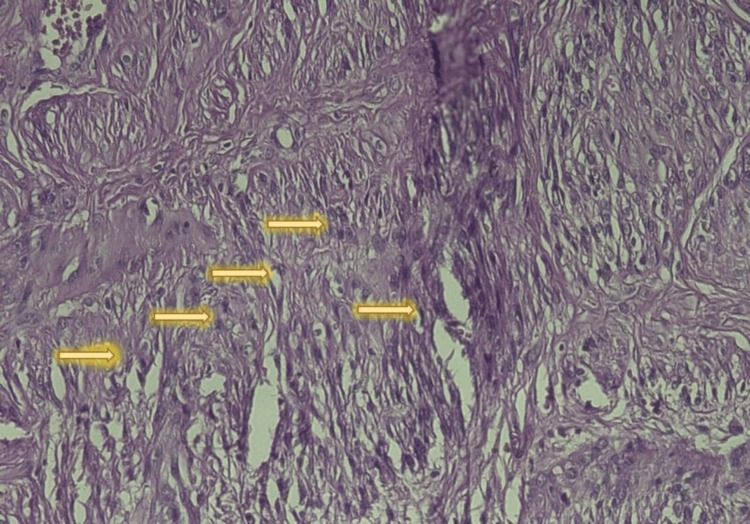
Photomicrograph showing multiple mitotic figures < 10 mitosis/10 HPF HPF: high-power field H&E: 40X

Intraoperatively, a solitary posterior intramural uterine fibroid of low vascularity was seen; it was well-circumscribed, fleshy, and measured 9×10 cm in the largest diameter. No area of hemorrhage nor necrosis was seen, and it did not show adherence or invasion to the surroundings. The mass was easily separated from its capsule and was sent for histopathology, see Figure 2. The surgery and the postoperative period went smoothly. We did not need a blood transfusion, and the patient was discharged home. The histopathological report confirmed the presence of uterine smooth muscle tumors of uncertain malignant potential (STUMP), see Figures 3-4. A total abdominal hysterectomy with ovarian preservation was performed after one month of initial surgery. It went uneventfully, with a normal postoperative course. The histopathology revealed normal uterine tissue with a small fibroid. At two months following the surgery, the patient is well and shows no signs of recurrence. She was advised to have regular surveillance visits due to the risk of late recurrence, as we already followed her up for two months.

## Discussion

The current case had no risk factors for having STUMPs. The presentation was typical of a case with fibroids. Neither the preoperative investigations nor intraoperative findings gave a hint of the diagnosis. Only a histopathology report confirmed the case, consistent with earlier reports [4,5]. This case was unique because it failed to respond to the Zoladex implant despite the patient's adherence to therapy, which underscores a more aggressive neoplastic process. The differential diagnosis includes:

Leiomyosarcomas: A malignant neoplasm of the hollow organ's smooth muscle fiber, including the uterus. They are typically unresponsive to hormones and are unaffected by Zoladex [1].

STUMPs: They are relatively resistant to hormonal therapy, including Zoladex [5].

Other leiomyoma sub-groups, including atypical leiomyoma and cellular leiomyoma [2].

STUMPs impose diagnostic and therapeutic challenges due to their unpredictable course. We have summarized their main clinical, laboratory, imaging, and intraoperative findings in Table 1.

**Table 1:** A summary of the clinical, biochemical, radiological, and intraoperative findings of cases with STUMPs STUMP: smooth muscle tumor of undetermined malignant potential

Category	Parameters checked	Ref.
Clinical history	Clinical picture. STUMP may be: Symptomatic - mostly abnormal uterine bleeding, chronic pelvic pain, mass sensation, pressure symptoms, and anemia; Asymptomatic - incidentally discovered in a routine investigation or infertility workup. Risk factors for STUMP. Older age women (especially perimenopausal); higher body weight; Black race; Expedient growth of the tumor.	[3]
Biochemical test	Increased levels of (LDH); were suggestive of tumor risk of recurrence and metastasizes. Thus, it may help in patients' risk categorization and guide treatment decisions and follow-up.	[6]
Imaging test	Ultrasound and MRI both share common features. Some suggested that a single mass, with a free fluid level with the absent acoustic shadowing, was seen in STUMPs. Doppler showed higher enhancement in STUMP cases owing to higher tumor vascularization. Others declared that on MRI, they show low-homogenous intensity in areas of T2. The use of contrast-enhanced MRI was helpful too.	[7,8]
Intraoperative	They tend to have larger sizes than fibroids, which has a worse prognosis for the patients. Sometimes, it exhibits irregular contour or may show a gross area of hemorrhage or necrosis. They sometimes adhere to or invade adjacent tissue.	[1]

The histopathological presentation of STUMP is quite diverse. Stanford’s criteria were used to establish their diagnosis. Characteristically, they show atypical smooth muscle tumor features, not meeting the criteria for leiomyoma or leiomyosarcoma [2]. Akbarzadeh et al. tested the role of immune-histochemical biomarkers in distinguishing STUMPs from fibroids. Their result showed that no single immune-histochemical biomarker is efficient enough to differentiate STUMPs [9]. They advised combining two biomarkers (P16 and Bcl-2) to improve the diagnostic efficacy.

Hysterectomy is the gold standard for managing STUMPs along with oophorectomy. Ovarian preservation is possible based on the women's special consideration [4]. Myomectomy alone is possible for women who wish to retain their fertility. Those cases attained successful pregnancies in 53% of the cases [10]. Interestingly, the recurrence risk for hysterectomy and myomectomy are the same, but the latter needs close patient follow-up [5]. The riskiest pathological signs for recurrence are the number of mitotic figures, cellular atypia degree, necrosis, vascular invasion, and absence of tumor-free margins [9]. As for the clinical risk factors for STUMP recurrence, some researchers have reported an association with age. The younger the patient is, the higher the recurrence risk [11]. Others suggested that having an intramural tumor increases the odds for recurrence up to (5.6) vs. subserosa tumors. The surgery type, smoking, and race were of no value in recurrence risk [5]. Adjuvant therapy's role is controversial. Data are scarce; some cases receive hormonal or chemotherapy while others receive radiotherapy [12]. Long surveillance is recommended by physical examination, imaging test, and serum biomarkers if they were initially raised. STUMP tends to recur up to 3.75 years following the primary surgery, with a total incidence of 7-14%, the highest for local recurrence [13]. Ideally, cases should be checked twice annually for the first five years and later annually. The STUMP prognosis is good; the five-year survival ranges from 60-90% [3,5].

## Conclusions

Managing STUMPS can be very challenging. They do not possess the characteristics of either a benign or malignant tumor. While clinical presentation, imaging modalities, and laboratory investigations may assist in diagnosing STUMP, histopathologic examination following hysterectomy or myomectomy is the definitive method for ultimate diagnosis.
